# Ciliostasis of airway epithelial cells facilitates influenza A virus infection

**DOI:** 10.1186/s13567-018-0568-0

**Published:** 2018-07-18

**Authors:** Yuguang Fu, Jie Tong, Fandan Meng, Doris Hoeltig, Guangliang Liu, Xiangping Yin, Georg Herrler

**Affiliations:** 1 Institute of Virology, University of Veterinary Medicine Hannover, Foundation, Hannover, Germany; 2 Lanzhou Veterinary Research Institute, State Key Laboratory of Veterinary Etiological Biology, Chinese Academy of Agricultural Sciences, Lanzhou, Gansu Province China; 3 Clinic of Swine and Small Ruminants and Forensic Medicine and Ambulatory Service, University of Veterinary Medicine Hannover, Foundation, Hannover, Germany

## Abstract

Porcine precision-cut lung slices (PCLS) were used to analyze the effect of the ciliary activity on infection of airway epithelial cells by influenza viruses. Treatment of slices with 2% NaCl for 30 min resulted in reversible ciliostasis. When PCLS were infected by a swine influenza virus of the H3N2 subtype under ciliostatic conditions, the viral yield was about twofold or threefold higher at 24 or 48 h post-infection, respectively, as compared to slices with ciliary activity. Therefore, the cilia beating not only transports the mucus out of the airways, it also impedes virus infection.

## Introduction, methods and results

The high frequncy of respiratory infections [1] indicates that the respiratory tract is a portal of entry that is frequently used by pathogenic microorganisms to enter a mammalian host. The continuous uptake of air and the large surface area make the airway system vulnerable by environmental factors. To prevent the detrimental effect of harmful substances, the respiratory tract is equipped with a mucociliary clearance system which is based on the function of specialized epithelial cells [reviewed in 2, 3]. Mucins released from mucus-producing cells form a viscous mucus-layer that entraps foreign particles, including viruses and bacteria. The removal of the entrapped material is accomplished by another type of specialized cells that are equipped with cilia. The cilia are surrounded by a fluid of low viscosity that allows their coordinated movement. As a result of this ciliary activity, the mucus layer that is floating on top of the periciliary layer is transported out of the airway.

We were interested to know whether the protective effect of the mucociliary clearance system is solely dependent on the mucus layer or whether the ciliary activity itself—apart from its mucus transport function—is also impeding infection by pathogens. For this purpose, we used swine influenza virus of the H3N2 subtype because of its superior growth properties in PCLS [4] to analyze the infection of differentiated respiratory epithelial cells by influenza virus in the absence and presence of ciliostatic conditions.

### Reversible ciliostasis in differentiated airway epithelial cells

To analyze the effect of ciliostasis on virus infection, we chose precision-cut lung slices as a culture system for differentiated airway epithelial cells. They were prepared as reported previously [5, 6]. Briefly, the cranial, middle, and intermediate lobes of fresh lungs of three-month-old clinically healthy pigs were obtained from the Clinics for Swine and Small Ruminants and Forensic Medicine and Ambulatory Service at the University of Veterinary Medicine, Foundation, Hannover, Germany. Prior to organ removal the pigs were euthanized by intravenous application of 80 mg/kg pentobarbital (Euthadorm^®^, Co. CP Pharma GmbH, Burgdorf, Germany). The lobes were filled with 37 °C warm low-melting agarose (AGAROSE LM; GERBU, Heidelberg, Germany). After solidification, cylindrical portions containing a bronchial airway were stamped out by using an 8-mm tissue coring tool. Using the Krumdieck tissue slicer (TSE systems, model MD4000-01, Bad Homburg, Germany), slices of about 250 μm thickness were generated. PCLS were transferred to 24-well plates (one slice/well) and maintained in 1 mL of RPMI 1640 medium (Genaxxon, Germany) supplemented with antibiotics and antimycotics. Slices were monitored under the light microscope (Zeiss Axiovert 35) for ciliary activity. For experiments, only slices were chosen in which the ciliary activity was retained on the whole luminal surface of the airway visible under the microscope (designated as 100% activity).

**Figure 1:**
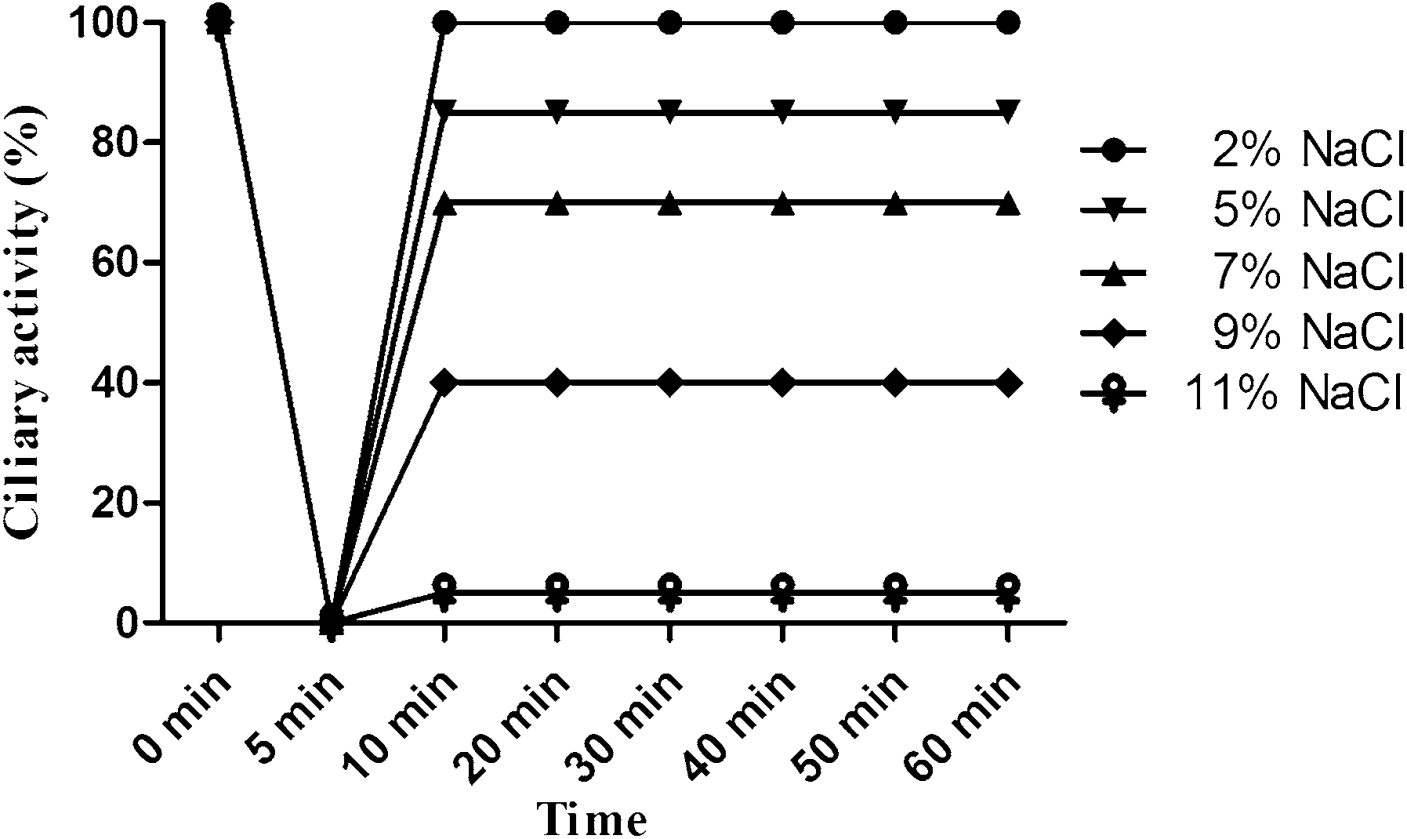
**Effect of hypertonic NaCl on ciliary activity of PCLS.** PCLS were treated with sodium chloride at different concentrations. When cilia had stopped beating, the culture medium was replaced by fresh medium without hypertonic sodium chloride. PCLS were monitored under the microscope for ciliary activity.

**Figure 2:**
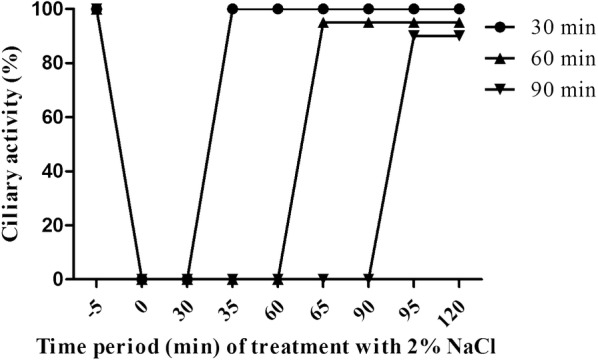
**The tolerance of PCLS under 2% NaCl condition.** PCLS were treated with 2% NaCl for different time period. At the indicated times, medium containing 2% NaCl was replaced by physiological medium. The ciliary activity was determined by microscopic inspection of the slices.

To induce ciliostasis, we applied hypertonic salt concentrations [7]. Porcine PCLS were treated with medium containing NaCl at the indicated concentrations. After incubation at 37 °C for the times indicated, the hypertonic medium was replaced by fresh medium immediately. As shown in Figure 1, treatment of PCLS with any of the different hypertonic salt concentrations for 5 min resulted in complete ciliostasis. When slices were returned to physiological medium, ciliary activity was recovered to some extent depending on the salt concentration used to induce ciliostasis. Full recovery of the ciliary activity—i.e. the whole luminal surface visible under the microscope showed cilia beating—was observed only with slices treated with 2% NaCl. Therefore, the following experiments were performed under this experimental condition. Next we analyzed which time period of treatment with high salt was tolerated by the slices without affecting the recovery of the ciliary activity. As shown in Figure 2, when PCLS were treated with 2% NaCl for 30, 60 and 90 min, ciliary activity was recovered to values of about 90% or somewhat higher. Recovery of the cilia beating on the whole luminal surface visible under the microscope was detected only when the salt treatment was as short as 30 min. Therefore, a short adsorption period was chosen for the infection experiments described below.

### Infection of PCLS under ciliostatic conditions

Swine influenza virus of the H3N2 subtype (A/sw/Herford/IDT5932/2007) was provided by Michaela Schmidtke, University of Jena, Germany. Virus was propagated in Madin-Darby canine kidney (MDCK) cells that were cultured with Eagle’s minimal essential medium (EMEM) containing 10% fetal bovine serum (Sigma, USA). Infected cells were incubated in EMEM supplemented with acetylated trypsin (Sigma-Aldrich, Munich) 1 μg/mL. After 72 h, the supernatant was harvested and, after removal of cell debris by centrifugation at 2000 × *g* for 10 min, virus was stored at −80 °C. Prior to infection of PCLS, we analyzed whether treatment with high salt affects the infectivity of influenza viruses. Virus was incubated with medium in the presence or absence of 2% NaCl for 30 min and then subjected to a plaque assay with MDCK cells as described previously [8, 9]. Both preparations were found to have the same titer of infectious virus (not shown) indicating that the high salt treatment did not affect the infectivity of the influenza viruses.

**Figure 3:**
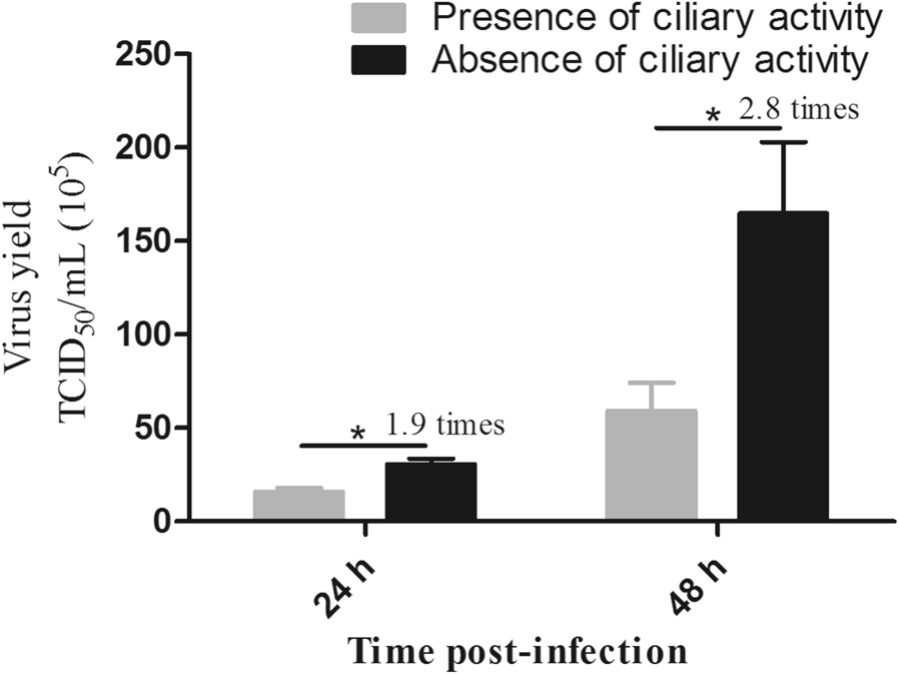
**Effect of ciliostatic conditions on the infection of PCLS by influenza virus.** PCLS were infected with H3N2 subtype of swine influenza virus at an infectious dose of 10^4^ TCID_50_ per slice in the presence or absence of the ciliary activity during the attachment step. After removal of the inoculum, cells were incubated under physiological conditions. Supernatants were collected at 24 and 48 hpi and analyzed for infectivity by TCID_50_ assay.

Porcine PCLS were infected with the H3N2 subtype of swine influenza virus at an infectious dose of 10^4^ TCID_50_ per slice for 20 min in the presence or absence of 2% NaCl, i.e. in the presence or absence of the ciliary activity. Supernatants were collected at different time points post-infection and stored at −80 °C. Viral titers of samples were determined by endpoint dilution titration on MDCK cells as described previously [10]. As shown in Figure 3, at 24 hpi, the virus titer was about twofold higher when PCLS were infected under ciliostatic conditions compared to infection at physiological conditions. At 48 hpi, the difference was about threefold. This result indicates that infection of differentiated airway epithelial cells by H3N2 influenza virus is more efficient in the absence of ciliary activity.

## Discussion

The role of the ciliary activity in transporting mucus with entrapped foreign substances out of the airways is a well-recognized feature of the mucociliary clearance system [2, 3]. We have addressed the question whether the protective role of the cilia beating is restricted to the mucus transport function or whether it has an additional effect on infection e.g. by impeding the attachment process of viruses. Even in the absence of mucus, the constant movement of the cilia may affect the viruses when they try to get access to cell surface. Precision-cut lung slices are a convenient culture system to examine the importance of the ciliary activity, because the cilia beating can be easily monitored by microscopic inspection. In this way we could determine conditions for reversible ciliostasis that are applicable to influenza virus infections. We indeed found that infection of porcine PCLS by a swine influenza virus of the H3N2 subtype was facilitated under ciliostatic conditions. A two- or threefold increase in the virus yield may appear to be not very impressive. However, this difference will be increased when infection involves more replication cycles. The success of a pathogen to induce an infection not only depends on the ability to initiate an infection but also on the efficiency. In this respect, the ciliary activity may help to prevent a number of infections.

It should be noted that we chose a microorganism that is very efficient in the attachment to target cells. Binding of influenza viruses to sialic acid containing receptors is readily observed after incubation for 20 min. Viruses or bacteria that attach to specific protein receptors are usually incubated with host cells for 2 h or more. It will be interesting to find out whether in such cases the impeding effect of the ciliary activity is increased.

For convenience, we chose hypertonic salt conditions to induce reversible ciliostasis. Influenza viruses were found not to be affected in their infectivity by high salt treatment. By contrast, *Streptococcus suis* stopped to amplify when treated with 2% NaCl. In such cases, one may use pharmacological substances to induce reversible ciliostasis [11, 12].

## References

[1] Monto AS, Malosh RE, Petrie JG, Thompson MG, Ohmit SE (2014). Frequency of acute respiratory illnesses and circulation of respiratory viruses in households with children over 3 surveillance seasons. J Infect Dis.

[2] Ganesan S, Comstock AT, Sajjan US (2013). Barrier function of airway tract epithelium. Tissue Barriers.

[3] Tilley AE, Walters MS, Shaykhiev R, Crystal RG (2015). Cilia dysfunction in lung disease. Annu Rev Physiol.

[4] Meng F, Punyadarsaniya D, Uhlenbruck S, Hennig-Pauka I, Schwegmann-Wessels C, Ren X, Dürrwald R, Herrler G (2013). Replication characteristics of swine influenza viruses in precision-cut lung slices reflect the virulence properties of the viruses. Vet Res.

[5] Goris K, Uhlenbruck S, Schwegmann-Wessels C, Köhl W, Niedorf F, Stern M, Hewicker-Trautwein M, Bals R, Taylor G, Braun A, Bicker G, Kietzmann M, Herrler G (2009). Differential sensitivity of differentiated epithelial cells to respiratory viruses reveals different viral strategies of host infection. J Virol.

[6] Punyadarsaniya D, Liang CH, Winter C, Petersen H, Rautenschlein S, Hennig-Pauka I, Schwegmann-Wessels C, Wu CY, Wong CH, Herrler G (2011). Infection of differentiated porcine airway epithelial cells by influenza virus: differential susceptibility to infection by porcine and avian viruses. PLoS One.

[7] Boek WM, Keleş N, Graamans K, Huizing EH (1999). Physiologic and hypertonic saline solutions impair ciliary activity in vitro. Laryngoscope.

[8] Vietmeier J, Niedorf F, Bäumer W, Martin C, Deegen E, Ohnesorge B, Kietzmann M (2007). Reactivity of equine airways: a study on precision cut lung slices. Vet Res Commun.

[9] Yang W, Punyadarsaniya D, Lambertz RL, Lee DC, Liang CH, Höper D, Leist SR, Hernández-Cáceres A, Stech J, Beer M, Wu CY, Wong CH, Schughart K, Meng F, Herrler G (2017). Mutations during the adaptation of H9N2 avian influenza virus to the respiratory epithelium of pigs enhance sialic acid binding activity and virulence in mice. J Virol.

[10] Van Rikxoort M, Michaelis M, Wolschek M, Muster T, Egorov A, Seipelt J, Doerr HW, Cinatl JJ (2012). Oncolytic effects of a novel influenza A virus expressing interleukin-15 from the ns reading frame. PLoS One.

[11] Boek WM, Romeijn SG, Graamans K, Verhoef JC, Merkus FW, Huizing EH (1999). Validation of animal experiments on ciliary function in vitro. I. The influence of substances used clinically. Acta Otolaryngol.

[12] Boek WM, Romeijn SG, Graamans K, Verhoef JC, Merkus FW, Huizing EH (1999). Validation of animal experiments on ciliary function in vitro. II. The influence of absorption enhancers, preservatives and physiologic saline. Acta Otolaryngol.

[13] European convention for the protection of vertebrate animals used for experimental and other scientific purposes. http://conventions.coe.int/Treaty/en/Treaties/Html/123.htm. Accessed 01 Jan 1991

[14] Protocol of amendment to the European convention for the protection of vertebrate animals used for experimental and other scientific purposes. http://conventions.coe.int/Treaty/en/Treaties/Html/170.htm. Accessed 02 Dec 2005

